# Opportunities for Epidemiological Data Collection in Dental Practices: A Thematic Analysis of Dutch Dentists’ Views

**DOI:** 10.1016/j.identj.2025.104023

**Published:** 2025-11-12

**Authors:** Joost C.L. den Boer, Maya Schulpen, Agatha W. van Meijeren-van Lunteren, Peggy C.J.M. van Spreuwel, Babette Everaars, Josef J.M. Bruers

**Affiliations:** a Department of Oral Public Health, Academic Centre for Dentistry Amsterdam (ACTA), University of Amsterdam and Vrije Universiteit Amsterdam, Amsterdam, The Netherlands; b Royal Dutch Dental Association (KNMT), Utrecht, The Netherlands; c National Institute for Public Health and the Environment (RIVM), Bilthoven, The Netherlands; d Research group Innovation in Preventive Healthcare, HU University of Applied Science, Utrecht, The Netherlands

**Keywords:** Ral health, Dental clinics, Epidemiologic methods, Dentist, Data collection, Electronic health records, Qualitative research

## Abstract

**Introduction and aims:**

Epidemiological oral health data are essential for informing public health policy and evaluating care. In the Netherlands, OrangeForce and the Oral Health Monitor seek to resume data collection by extracting information from dental practices using both automated (ie, software-based extraction) and manual (ie, user-entered in web-based forms) methods. As the success of these methods depends on cooperation from providers, this study aimed to explore the perspectives of Dutch dentists on collecting oral health data from practices, focussing on automated and manual data collection.

**Methods:**

Thirteen dentists were individually interviewed between May-December 2024 using a predefined topic list. The initial three participants were purposively sampled; others were approached to ensure variation in background characteristics. The interviews were thematically analysed using Atlas.ti.

**Results:**

The dentists – assisted by a dental assistant – recorded most data directly during check-ups. Recorded data varied considerably in both content and method of documentation. Although the dentists generally supported data collection, they noted that more consistent and structured recording would be required for automated methods. Manual collection was perceived as time-consuming, although clear instructions could mitigate this. Eventually, the decision to participate in manual data collection largely depended on the required workload and time investment. Benchmark feedback could serve as a key incentive but is informative only with sufficiently large samples per dentist.

**Conclusions:**

Dutch dentists widely acknowledged the importance of epidemiological oral health data collection but identified several practical challenges. Currently, manual data collection via web-based forms seems more feasible than automated registration, which requires technical improvements and workflow changes. Clear instructions and the involvement of oral care providers in the development of the data collection procedures are necessary to improve data quality and manage research burden. Further advancement of dental software systems is needed to facilitate structured data collection in the future.

## Introduction

Epidemiological data can provide insight into population oral health, inequalities and related trends.^1^^,^^2^ This information supports development, implementation, and evaluation of public health interventions and healthcare systems. In 2012, the Health Council of the Netherlands emphasised the need for more knowledge about the oral health of the Dutch population. To address this, they recommended establishing a national monitoring system to collect data on oral health, with particular attention to regional differences, vulnerable groups, public knowledge about the prevention of oral diseases, and the (financial) accessibility of oral care.^3^ The collection of epidemiological data, however, requires human and financial resources, which are not always available.^4^ Although technological advances (eg, electronic health records kept by dental practices) have increased the possibilities for data collection in recent years, the quality of the collected data remains a challenge.^5^^,^^6^ Additionally, there are challenges related to data availability, stakeholder acceptance, expertise, and privacy.^7^

The most recent large-scale cross-sectional collection of oral health data in the Netherlands took place in 2013 for adults and in 2017 for the youth.^8^^,^^9^ In recent years, two initiatives have been launched to resume this data collection: OrangeForce (ORal ANd GEneral For OldeR People’s CarE) and the Oral Health Monitor of the National Institute for Public Health and the Environment (RIVM).^10^^,^^11^ OrangeForce was established within the ORANGEHealth consortium to support the integration of oral healthcare into the broader healthcare system. This is pursued by developing guidelines for both dental practices and broader healthcare settings, and by promoting collaboration between dental professionals and primary care providers.^11^ Additionally, efforts are being made to enhance the accessibility of patient data for research and to develop diagnostic tools for the early detection of diseases. As part of this initiative, work is undertaken to unlock and integrate oral health data, including an assessment of the feasibility of extracting data from patient records in Dutch dental practices. In 2023, the RIVM developed a set of indicators in collaboration with a group of experts to monitor oral health.^12^ As part of the monitor, an interactive dashboard was developed and annual updates will be provided together with yearly publication of reports.^13^ To date, data are available for a selection of indicators, based on self-report and declarations. However, for the remaining 11 indicators, clinical data are required that are not yet available.

Patient records from dental practices may serve as a valuable source of data on the clinical oral health outcomes that are currently not available for the Oral Health Monitor. According to data from Statistics Netherlands, approximately 80% of the Dutch population visits the dentist annually.^14^ This indicates that dental practices could, in theory, provide access to relevant clinical data for the majority of the population. It is, however, not known whether such data are consistently and systematically recorded in a way that allows for meaningful analysis. By contrast, in primary healthcare, the Netherlands has established data registries that support health services research at national and regional levels.^15^

Given their shared objectives, the Oral Health Monitor and OrangeForce have joined forces. To ensure short-term feasibility in light of financial constraints, the initial focus was on identifying a method to rapidly collect existing data from routine practice, specifically, data already recorded for clinical purposes and readily shareable by oral healthcare professionals. As part of this effort, Vertimart – market leader in dental software and partner in OrangeForce – committed to implementing a functionality in Exquise Next Generation that enables the anonymous and automated transfer of the desired patient data, hereafter referred to as automated data collection. Pending its implementation, a pilot data collection strategy was introduced in the form of ‘*Data Collection Weeks’.* During these weeks, participating oral healthcare providers will manually enter specific data directly into a web-based form for a small sample of patients across different age groups. This user-entered form of data entry is referred to as manual data collection.

As both data collection methods require extra effort from providers, understanding their perspectives is essential for effective implementation, taking into account regional and field-specific differences within Dutch oral healthcare.^7^^,^^16^ To date, no research has been conducted on this topic among Dutch dental care providers. Therefore, this study aimed to explore the perspectives of Dutch dentists on the collection of oral health data from oral healthcare practices, with a special focus on the implications of automated and manual data collection methods, the latter will be used in the Data Collection Weeks.

## Methods

### Research instrument

Data for this study were collected using individual semi-structured interviews with dentists, which were analysed through thematic analysis. All interviews were conducted remotely via Zoom by experienced interviewers. A topic list was developed as a framework for the interviews. The topic list is available in Supplement 1 in both the original Dutch and a translated English version produced through a forward–backward translation process. The topic list covered the following subjects: availability of oral health and treatment data, perceptions about recording these data, possibilities for collecting data from dental practices, willingness to cooperate, and interest in benchmark data as an incentive for participation.

### Data collection

A total of 47 dentists were contacted. Potential participants first received an email with information about the study’s topic and design, followed by a phone call inviting them to participate voluntarily. The interviews were conducted by three researchers: JdB, JB and PvS. The first three interviews (May 2024) involved dentists from the Data Stations Project network of the Royal Dutch Dental Association (KNMT), who were selected through purposive sampling to ensure familiarity with the subject matter.^17^ For the remaining interviews, which were conducted between October and December 2024, a random group of dentists was approached. Efforts were made to ensure variation in gender, age, province of establishment, place of graduation, and work situation. Invitations were sent out in stages until interviews yielded no new insights and data saturation appeared to be achieved; interviews already scheduled continued as planned.

### Data analysis

The data were analysed using thematic analysis with the software package Atlas.ti (version 25, Scientific Software Development GmbH, Berlin, Germany) following the approach of Braun and Clarke.^18^ The interviews were transcribed verbatim using Amberscript (https://www.amberscript.com) and the resulting transcripts were reviewed by JdB, who was also primarily responsible for the analysis, working in coordination with MS. Familiarisation with the data occurred by performing part of the interviews and checking the transcripts (JdB) and by summarising all interviews (MS). The summaries were also sent to participants for a member check to confirm that the data accurately reflected their perspectives, providing internal validation. The initial codes were manually assigned by JdB in coordination with MS. Subsequently, JdB and MS jointly identified the underlying themes and refined them through discussion until consensus was reached. Taking the original topic list as a starting point, the themes were reorganised in light of patterns that emerged during the analysis. Participant quotes are included in the Results section to substantiate the identified themes. Since the interviews were conducted in Dutch, these quotes were translated to English using forward-backward translation. An overview of the original Dutch quotes and their English translations is provided in Supplement 2.

### Ethics statement

In the information letter and during the phone invitation, the study design was clearly explained. It was explicitly stated that the interview would be recorded and that participation was voluntary. This was reiterated at the start of the interview, and informed consent was formally recorded.

The study protocol was reviewed by the Ethical Review Board (Ethische Toetsingscommissie) of the Academic Centre for Dentistry Amsterdam (ACTA) and approved on March 28, 2024 (reference number: 2024-80060).

## Results

### Research population

Of the 47 invited dentists, five declined by email. The remaining 42 were approached by telephone, but 14 could not be reached after several attempts. Of the 28 dentists contacted, 13 declined (mostly due to time constraints) and two cancelled after initially agreeing, with no opportunity to reschedule. In total, 13 dentists were interviewed. Table 1 shows that variation was achieved in terms of gender (7 women and 6 men), age (ranging from 30 to 67 years), region of establishment (9 of the 12 Dutch provinces were covered), place of graduation (participants included graduates of all three current dental schools in the Netherlands and one now-discontinued dental school; one dentist obtained her degree abroad), and work situation (11 practice owners and 2 non-practice owners).

**Table 1:** Background characteristics of the interviewed dentists.

Respondent no.	Gender	Age	Province of establishment	Place of graduation	Work situation
1	Male	60	Utrecht	Utrecht	Practice owner
2	Female	30	Groningen	Groningen	Non-practice owner
3	Female	59	Noord-Holland	Amsterdam	Practice owner
4	Male	37	Zuid-Holland	Nijmegen	Practice owner
5	Female	38	Noord-Holland	Groningen	Practice owner
6	Male	42	Drenthe	Groningen	Practice owner
7	Female	67	Gelderland	Germany	Practice owner
8	Male	39	Overijssel	Nijmegen	Practice owner
9	Female	59	Noord-Brabant	Amsterdam	Practice owner
10	Male	37	Flevoland	Amsterdam	Practice owner
11	Male	55	Noord-Brabant	Nijmegen	Practice owner
12	Female	45	Noord-Brabant	Nijmegen	Non-practice owner
13	Female	42	Groningen	Groningen	Practice owner

### Themes

The following themes were derived from the analysis: [1] method of data recording in practice, [2] attitude towards data recording, [3] availability of epidemiological data, [4] conditions for participation in epidemiological data collection, and [5] value of benchmark data.

*Theme 1: Method of data recording in practice*

Most oral health data were recorded during the dental check-up, often at the same time as the actual examination took place: the assistant directly added the dentist’s observations to the patient’s record. The dentist however remained responsible for the accuracy of the patient records and generally checked them, either immediately after the appointment or later that day. Some dentists mentioned recording all data themselves. An exception was the periodic periodontal score (PPS,^19^), the Dutch standard for recording periodontal screening, which was often recorded by dental hygienists or preventive assistants.

*Theme 2: Attitude towards data recording*

For dentists, it was important that the recorded oral health data were useful for treatment. Some respondents expressed interest in expanding the range of routinely recorded data, particularly for specific patient groups, but emphasized that such recording must remain feasible in daily practice.

*1 “With our Mandibular Advancement Device patients/sleep apnea patients, we have a whole list of things that we go through during the first visit. You could maybe do something a bit more extensively for a check-up as well. But of course, it also has to remain manageable.” (respondent 13)*

While dentists valued accurate data recording in the context of their own care provision, they especially emphasised its potential benefits for collaboration, frequently viewing their own approach as the standard. Adjusting to a new way of recording would require a time investment for both dentists themselves as well as their supporting staff. While the software’s capabilities were appreciated, not all features are currently being used. Some dentists admitted that they were probably not familiar with all features available.

*2. “Well, I believe that it offers more possibilities than what I am currently making use of.” (respondent 5)*

Recording data during the check-up was seen as the least prone to errors. It was important for the dentists to explain to the patient what was being recorded and why. Although standardized recording would take more time, the dentists recognized that it could help reduce errors, for example, when non-standardized entries are unclear or misinterpreted. However, they also noted that standardized forms may not always accommodate all clinical situations.

*3 “Well, that functions incredibly easily of course, and although the selected item may not always be exactly correct, it is still very convenient to use.” (respondent 11)*

Finally, one dentist noted the potential of using artificial intelligence and speech recognition technologies for future data recording.

*Theme 3: Availability of epidemiological data*

Dentists reported documenting a wide range of patient information, including personal and medical information, dental history, current dental status, untreated caries (to be treated or monitored), caries risk, filled elements, materials used, PPS, other periodontal characteristics, self-care and oral hygiene, present removable appliances, present crowns, root canal treatments, notable findings, treatment plan, reasons for treatments, practitioner, and reasons for switching from a previous dentist. However, these data were neither always recorded consistently nor systematically documented in the same place in the patient file, while several respondents noted that automated data recording would require consistent and systematic input without omissions.

*4 “It really is my own way of recording data.” (respondent 2)*

Differences between dental software programs were another reason for the variation in the amount and method of recording:

*5 “That what you record is of course also very dependent on what is included in your software program.” (respondent 4)*

Variation in data was also attributable to differences of opinion on what should be recorded, further complicated by the fact that not everything can be scored equally well. Generally, variation within a practice was not considered a major issue, but some dentists viewed differences between practices as significant. As a result, discrepancies may arise in patient files when new patients bring records from their previous dentist.

*Theme 4: Conditions for participation in epidemiological data collection*

Most dentists expressed willingness to contribute to epidemiological data collection, though the perceived feasibility differed between automated and manual collection methods. The willingness partly depended on the anticipated time investment, with manual data collection requiring more time than automated methods. Also, the administrative burden and workload were significant factors in the decision to participate.

*6 “I do think that if you wanted to collect this systematically, and if that can’t be easily automated, then you would probably face a lot of resistance. Or at least in terms of time investment.” (respondent 10)*

A number of suggestions were made to limit the time investment required for manual data collection. For example, it was recommended to utilize the capabilities offered by the dental software to collect data that had already been recorded and to use standard formats as much as possible. Clear and concise instructions were seen as helpful in this process.

*7 “I think it is important that there are really good instructions with it” (respondent 11)*

According to some respondents, it would be possible for a dental assistant or another co-worker in the practice to record the data with proper instructions. However, one respondent pointed out that this should not take up too much time and financial resources. In this regard, it was also noted that, in case of manual data collection, the research should be announced in advance to enable the participating dentists to allocate the necessary time.

Furthermore, some dentists highlighted preconditions that the data collection must meet, such as privacy considerations, instructions for the random selection of patients, informed consent from the patient, and the necessity to calibrate the data of participating dental care providers. These conditions applied to both automated and manual data collection, although to varying extents. Generally, obtaining informed consent from patients was not considered problematic, nor was asking a few research-related questions. Nevertheless, it was noted that expecting more from patients could complicate the process. Finally, it was mentioned that it is important to recognize that data will only be collected from patients who visit dental practices, thereby excluding populations less likely to access oral healthcare.

*Theme 5: Value of benchmark data*

Several incentives were mentioned that could encourage dentists to participate in the research. Although financial compensation and additional training points for the Quality Register for Dentists were discussed, the most frequently mentioned incentive was the provision of benchmark data allowing dentists to compare their own patients with those of other participants. This type of information was generally regarded as interesting.

*8 “Yes, that would be interesting for me to look at. Are you performing well or are we on the right track? Or do we need to start doing things differently?” (respondent 8)*

However, many respondents pointed out that when data were collected manually for only a small number of patients, as suggested for the Data Collection Weeks, benchmark data would become less useful. In that case, it was appreciated if the results were actively shared with participants, allowing specific differences between patient groups to be explored in greater detail. There seemed to be no strong preferences regarding the presentation format or frequency of the feedback. Additionally, some negative aspects of benchmark data were noted. For example, some dentists might not feel motivated if comparisons showed that others were performing better. Furthermore, one dentist expressed concern about the potential use of benchmark data to monitor or regulate oral healthcare practices.

## Discussion

The aim of this qualitative study was to explore Dutch dentists’ opinions on recording and collecting epidemiological oral health data. The collection of these data is a key objective in both OrangeForce and the Oral Health Monitor. These initiatives aim to use such data for mapping the oral health of the Dutch population (as emphasised in the Oral Health Monitor) and for supporting data exchange with other healthcare providers (as emphasised in OrangeForce). Specific attention was given to the potential of automated processes in dental administration software for large-scale data collection as well as the possibility of manual submission of data for a limited number of patients per practice.

Both automated (software-based extraction) and manual (user-entered in web-based forms) data collection methods have distinct advantages and limitations. A key advantage of automated data collection is the ability to gather large volumes of data with minimal time investment for oral healthcare providers. To achieve this, the appropriate functionalities must be integrated within dental software. This, however, depends on the willingness and capability of software suppliers to support standardized data entry. For successful adoption, dentists emphasized the importance of perceivable benefits for daily clinical and operational practice – such as facilitating the recording of process and outcome indicators – beyond solely serving epidemiological purposes.^20^ Beyond differences in feasibility automated and manual collection methods also differ in the structure of the resulting data. Manual methods produce simple, straightforward – also called flat – datasets, while automated extraction produces larger, more complex datasets requiring advanced analytical techniques.^21^^,^^22^

Furthermore, this study indicates that merely providing standardized data collection options is insufficient. Clinical data are often incompletely and inconsistently recorded, limiting their suitability for automated extraction.^23^, ^24^, ^25^ Shifting data recording practices will require time, training, and changes in established workflows.^26^^,^^27^ Most current dental software programs were developed for administrative rather than diagnostic or research purposes, further complicating standardized data entry.^25^ Dentists in this study noted that adoption would be facilitated if software demonstrated clear clinical value, such as supporting diagnosis, monitoring outcomes, and sharing information with other colleagues. The upcoming European Health Data Space (EHDS), may promote interoperable and reusable health data by introducing EU-wide standards for secure data exchange and secondary scientific use.^28^ Achieving consistency in standardized data recording is challenging due to individualized workflows and evolving routines within and between practices.^25^^,^^26^ Speech recognition may be a promising solution, as it aligns with current practice, improves accuracy and efficiency, and promotes the use of standardized terminology.^29^, ^30^, ^31^

Given the current limitations of automatic data extraction, participants perceived manual data collection as a feasible short-term alternative due to its ease of implementation and flexibility in targeting specific data. However, the required time investment limits data volume. Dutch research on quality indicators in oral healthcare showed that dentists were apprehensive of the administrative burden of quality measurement, along with being monitored.^32^ While respondents expressed willingness to make this investment, actual participation may be lower, as seen in a similar design by Bots-van ’t Spijker and colleagues.^33^ To ensure the feasibility of the manual data collection implemented during the ‘*Data Collection Weeks’*, it is important to minimise the research burden on dentists by keeping the sample size and the recorded data per patient manageable, using familiar instruments and providing clear instructions and support.^34^^,^^35^ While small samples may introduce selection bias and limit benchmarking potential, clear selection criteria can mitigate bias.^36^, ^37^, ^38^ Despite these limitations, the approach remains suitable for studying a small number of patients per practice during the *‘Data Collection Weeks’*, as the resulting data do not have to fully reflect the broader patient population.

It is important to realize that, regardless of collection method, data are limited to dental attenders, excluding about 20% of the Dutch population who do not visit dental practices for various reasons.^39^ This group often includes individuals with lower socioeconomic status, who are at higher risk for oral health problems.^14^^,^^40^ As socioeconomic status is associated with oral health, the absence of this group may lead to an underestimation of oral health problems in the general population.^9^^,^^41^ Alternative strategies are needed to reach this group and reduce bias in population estimates. Engaging individuals with lower socioeconomic status in epidemiological research has proven challenging in the past.^9^ Still, data collected directly from oral healthcare practices can offer a valuable and practice-based perspective. At the same time, such data may be influenced by variations in practice routines, staff roles, and recording habits.^42^

### Strengths and limitations

The semi-structured interview format allowed dentists to describe in detail and in their own words what they record, why they do so, and how they perceive the use of these data. This has resulted in a comprehensive picture, clearly highlighting both the possibilities and barriers of automated and manual data collection. Naturally, a group of thirteen dentists is too small to determine how widespread these views are within the entire population of 9555 dentists in the Netherlands.^43^ However, this research does provide a deeper understanding of the considerations that influence Dutch dentists in deciding whether to make practice-based data available for epidemiological research, including their motivation to participate and the obstacles they anticipate.

A limitation of this study is that only dentists were interviewed, which may have excluded perspectives from other oral healthcare professionals. In the Netherlands, 3900 dental hygienists are active, some of whom working in independent practices.^43^ These practices are directly accessible to patients and do not require referral from a dentist.^44^ If dental hygienists’ views on any of the studied topics happen to differ from those of dentists, their opinions may be underrepresented in this study as only dentists were interviewed. Additionally, dental assistants were also not included, even though they participated in recording patient data in the practices of several interviewed dentists and were, in some cases, identified as potential personnel responsible for the data collection process. Therein, the barriers, opportunities, and conditions they perceive may also be relevant. Finally, since the research was conducted within the context of two national Dutch projects, only dentists practising in the Netherlands were interviewed. Given the regional nuances that characterise oral healthcare in the Netherlands, this is a justifiable choice,^7^ though it does limit the generalisability to other countries. That said, the issues raised in this study are likely to be relevant beyond the national context, particularly in comparable Western European settings. The EHDS establishes regulations that apply across all countries in the European Union, making at least some of the challenges related to data collection for research purposes comparable in many of them.^28^

## Conclusions

Dutch dentists widely acknowledged the importance of collecting oral health data for epidemiological purposes but identified several practical challenges. Manual data collection is currently more feasible than automated extraction as technical capabilities are lacking and implementation would require significant workflow adjustments. To minimize research burden, clear instructions and integration with existing records are essential. Active involvement of oral healthcare providers in developing data collection instruments and procedures is crucial. Further advancement of dental software systems is needed to facilitate structured data collection in the future.

## Appendix Supplementary materials

Image, application 1

Image, application 2

## Footnotes

Supplementary material associated with this article can be found in the online version at doi:10.1016/j.identj.2025.104023.

## References

[1] Peres M., Antunes J., Watt R. (2020). The contribution of epidemiology to Oral Health Research. Oral epidemiology: a textbook on oral health conditions, research topics and methods.

[2] Veiga N., Coelho I. (2015). The importance of epidemiology in dental medicine. Journal of dental and oral health.

[3] Health Council of the Netherlands (2012).

[4] Strauss W., Ryan L., Morara M. (2010). Improving cost-effectiveness of epidemiological studies via designed missingness strategies. Stat Med.

[5] Kilgus T., Patecka A., Schurig T. (2024). Creating value from the secondary use of health data: international examples, best practices, and opportunities to scale. Communications of the Association for Information Systems.

[6] World Health Organization (2019).

[7] Zisis K., Pavi E., Geitona M., Athanasakis K. (2024). Real-world data: a comprehensive literature review on the barriers, challenges, and opportunities associated with their inclusion in the health technology assessment process. Journal of Pharmacy & Pharmaceutical Sciences.

[8] Schuller A., van Kempen I., Vermaire E. (2014).

[9] Schuller A., Vermaire E., van Kempen I., van Dommelen P., Verrips E. (2018). Kies voor Tanden 2017: Een onderzoek naar mondgezondheid en preventief tandheelkundig gedrag van jeugdigen. Hoofdmeting 2017, een vervolg op de reeks TJZ-en Kies-voor-Tandenonderzoeken.

[10] Everaars B., Baadoudi F., van den Ende C., Prusak A., Kuijpers T. (2023). Plan van aanpak monitor Mondgezondheid.

[11] Orange Health (2025). https://orangehealth.nl/orange-force/.

[12] Everaars B., Baâdoudi F., Van den Ende C., Kuijpers T. (2022).

[13] (2025). Monitor Mondgezondheid Bilthoven.

[14] Centraal Bureau voor de Statistiek (2024). https://opendata.cbs.nl/statline/#/CBS/nl/dataset/85463NED/table?ts=1742762560610.

[15] Bes J, Arslan I, Treurniet J (2025). Naar een lerend zorgsysteem voor de mondzorg.

[16] Cheng C., Messerschmidt L., Bravo I. (2024). A general primer for data harmonization. Sci Data.

[17] Den Boer J., Van der Sanden W., Bruers J. (2020). Developments in oral health care in the Netherlands between 1995 and 2018. BMC Oral Health.

[18] Braun V., Clarke V. (2006). Using thematic analysis in psychology. Qual Res Psychol.

[19] Van Strydonck D., Katsamakis S., Van der Weijden G. (2020).

[20] Righolt A., Sidorenkov G., Faggion C., Listl S., Duijster D. (2019). Quality measures for dental care: a systematic review. Community Dent Oral Epidemiol.

[21] Austin P., Goel V., Van Walraven C. (2001). An introduction to multilevel regression models. Can J Public Health.

[22] Fan J., Han F., Liu H. (2014). Challenges of big data analysis. Natl Sci Rev.

[23] Da Silva F., Firmiano C., de Alencar Júnior E. (2025). Challenges and advantages of implementing electronic dental records: a literature review. Research, Society and Development..

[24] Gurupur V., Vu G., Mayya V., King C. (2024). The need for standards in evaluating the quality of electronic health records and dental records: a narrative review. Big Data Cogn Comput.

[25] Gianfrancesco M., Goldstein N. (2021). A narrative review on the validity of electronic health record-based research in epidemiology. BMC Med Res Methodol.

[26] Abernethy A., Adams L., Barrett M., Bechtel C., Brennan P., Butte A. (2022). The promise of digital health: then, now, and the future. NAM Perspect.

[27] Volgenant C., Bras S., Persoon I. (2022). Facilitators and barriers to implementing sustainability in oral health care. Int Dent J.

[28] Marcus J., Martens B., Carugati C., Bucher A., Godlovitch I. (2022). The european health data space. Luxemburg Policy Department for Economic, Scientific and Quality of Life Policies.

[29] Johnson M., Lapkin S., Long V., Sanchez P., Suominen H., Basilakis J. (2014). A systematic review of speech recognition technology in health care. BMC Med Inform Decis Mak.

[30] Joseph J., Moore Z., Patton D., O’Connor T., Nugent L. (2020). The impact of implementing speech recognition technology on the accuracy and efficiency (time to complete) clinical documentation by nurses: a systematic review. J Clin Nurs.

[31] Kumar Y. (2024). A comprehensive analysis of speech recognition systems in healthcare: current research challenges and future prospects. SN Comput Sci.

[32] Righolt A., Duijster D., Smits K., Oerlemans A., Van der Wees P., Listl S. (2025). Stakeholders’ perspectives on quality measurement of oral health care in the netherlands: a qualitative study. Int Dent J.

[33] Bots-VantSpijker P., Van der Maarel-Wierink C., Schols J., Bruers J. (2022). Oral health of older patients in dental practice: an exploratory study. Int Dent J.

[34] Girard A., Dugas M., Lépine J., Carnovale V., Jalbert L., Turmel A. (2023). Strategies to engage family physicians in primary care research: a systematic review. J Eval Clin Pract.

[35] Ngune I., Jiwa M., Dadich A., Lotriet J., Sriram D. (2012). Effective recruitment strategies in primary care research: a systematic review. Qual Prim Care.

[36] Keeble C., Law G., Barber S., Baxter P. (2015). Choosing a method to reduce selection bias: a tool for researchers. Open J Epidemiol.

[37] Tripepi G., Jager K., Dekker F., Zoccali C. (2010). Selection bias and information bias in clinical research. Nephron Clin Practice.

[38] Wind A., Van Harten W. (2017).

[39] Steinvik L., Svartdal F., Johnsen J. (2023). Delay of dental care: an exploratory study of procrastination, dental attendance, and self-reported oral health. Dent J (Basel).

[40] Begovic S., Van der Heijden G., Listl S. (2023).

[41] De Abreu M., Cruz A., Borges-Oliveira A., Martins R., Mattos F. (2021). Perspectives on social and environmental determinants of oral health. Int J Environ Res Public Health.

[42] Brown L. (2015). Inadequate record keeping by dental practitioners. Aust Dent J.

[43] (2025). Werkers in de mondzorg Utrecht.

[44] Jongbloed-Zoet C., Bol-Van den Hil E., La Riviere-Ilsen J., Van der Sanden-Stoelinga M. (2012). Dental hygienists in The Netherlands: the past, present and future. Int J Dent Hyg.

