# Supplementary material for: Opportunities for Epidemiological Data Collection in Dental Practices: A Thematic Analysis of Dutch Dentists’ Views
Source: Int Dent J. 2025 Nov 12;76(1):104023. doi: 10.1016/j.identj.2025.104023 (PMC12657284; doi:10.1016/j.identj.2025.104023)
Supplement: Supplementary file 1 [file mmc1.docx]

| **Original topic list (Dutch)** | |
| --- | --- |
| 1 Beschikbaarheid van mondgezondheidsgegevens | a Welke gegevens worden bij elke patiënt standaard vastgelegd?  b Welke daarvan worden volgens vaste ‘afgesproken’ codering vastgelegd (gestructureerd in afspraken / bestaand format)?  c Welke daarvan worden in eigen ‘codering’ vastgelegd? |
| 2 Percepties over vastleggen van mondgezondheids- en behandelingsgegevens | a Op welk moment van het patiëntcontact c.q. de behandeling worden gegevens doorgaans vastgelegd?  b Wie is er binnen de praktijk verantwoordelijk voor de vastlegging van mondgezondheids- en behandelingsgegevens?  c Hoe grondig beschouwt u de vastlegging van patiënt- en behandelingsgegevens in uw werksituatie?  Is verbetering wenselijk?  d Hoe gestructureerd beschouwt u de vastlegging van patiënt- en behandelingsgegevens in uw werksituatie?  Is verbetering wenselijk? |
| 3 Mogelijkheden om mondgezondheids- en behandelingsgegevens te verzamelen vanuit praktijken voor epidemiologisch onderzoek c.q. uitwisseling ten behoeve van gezondheidsmonitoring | a Wat zijn de belangrijkste doelen en voordelen van het verzamelen van gegevens vanuit praktijken voor onderzoek en uitwisseling?  b Zijn er kansen hiervoor gezien het huidige proces van vastleggen van mondgezondheids- en behandelingsgegevens?  Zo ja, welke?  c Zijn er uitdagingen of beperkingen in het huidige proces van vastleggen van mondgezondheids- en behandelingsgegevens?  Zo ja, welke? |
| 4 Bereidheid om mondgezondheids- en behandelingsgegevens te verstrekken | a Zou u bereid zijn om mondgezondheidsgegevens aan te leveren voor onderzoek en uitwisseling?  Zo nee, waarom niet?  b Zo ja, zijn er specifieke obstakels of belemmeringen die u verwacht bij het aanleveren van gegevens voor onderzoek en uitwisseling?  c Wat zou er nodig zijn het aanleveren zo makkelijk mogelijk te maken  (bepaalde technologieën of hulpmiddelen die te benutten zijn voor het vastleggen en verzamelen van de gegevens)? |
| 5 Interesse in het ontvangen van terugrapportage of spiegelinformatie over de verstrekte gegevens | a Heeft u behoefte aan terugrapportage over de verstrekte gegevens?  Zo nee, waarom niet?  b Zo ja, hoe vaak zou u updates of feedback willen ontvangen over de door u en anderen verstrekte gegevens?  c In welke vorm zou u graag terugrapportage ontvangen (bijvoorbeeld rapporten, grafieken, feedbackgesprekken e.a.)? |
| 6 Wat verder ter tafel komt |  |

| **Translated topic list (English)** | |
| --- | --- |
| 1 Availability of oral health data | a Which data are routinely recorded for each patient?  b Which of these data are recorded using standardized coding, based on established conventions or existing structured formats?  c Which of these data are recorded using non-standardized or personal coding? |
| 2 Perceptions on recording oral health and treatment data | a At which stage of the patient consultation or treatment are data generally recorded?  b Within the practice, who is responsible for recording oral health and treatment data?  c How thorough do you consider the recording of patient and treatment data in your work environment?  Do you believe improvement is desirable?  d How structured do you consider the recording of patient and treatment data in your work environment?  Do you believe improvement is desirable? |
| 3 Opportunities for collecting oral health and treatment data from dental practices for epidemiological research and data exchange in support of health surveillance | a What are the main goals and benefits of collecting data from dental practices for research and exchange?  b Are there opportunities for this, given the current process of recording oral health and treatment data?  If so, what are they?  c Are there challenges or limitations in the current process of recording oral health and treatment data?  If so, what are they? |
| 4 Willingness to provide oral health and treatment data | a Would you be willing to provide oral health data for research and exchange?  If not, why not?  b If so, are there any specific obstacles or barriers you anticipate in providing data for research and exchange?  c What would be needed to make the provision of data as convenient as possible (certain technologies or tools that could be utilized for recording and collecting the data)? |
| 5 Interest in receiving feedback or benchmarking information about the provided data | a Would you like to receive feedback about the provided data?  If not, why not?  b If so, how often would you like to receive updates or feedback on the data provided by you and others?  c In what form would you prefer to receive feedback (e.g., reports, charts, feedback sessions, etc.) |
| 6 Any other matters that may arise |  |
