# Supplementary material for: Opportunities for Epidemiological Data Collection in Dental Practices: A Thematic Analysis of Dutch Dentists’ Views
Source: Int Dent J. 2025 Nov 12;76(1):104023. doi: 10.1016/j.identj.2025.104023 (PMC12657284; doi:10.1016/j.identj.2025.104023)
Supplement: Supplementary file 2 [file mmc2.docx]

|  | **Original quotes (Dutch)** | **Translated quotes (English)** | |
| --- | --- | --- | --- |
| 1 | Bij onze MRA patiënten/slaapapneu patiënten hebben we een heel lijstje van dingen die we het eerste bezoek even nalopen. Zo zou je dat voor een controle misschien ook wat uitgebreider kunnen doen. Maar goed, dat moet ook werkbaar blijven. | With our Mandibular Advancement Device patients/sleep apnea patients, we have a whole list of things that we go through during the first visit. You could maybe do something a bit more extensively for a check-up as well. But of course, it also has to remain manageable. |  |
| 2 | Nou, ik denk dat je wel dat er meer kan dan dat je dat ik steeds doe. | Well, I believe that it offers more possibilities than what I am currently making use of. |  |
| 3 | Nou dat werkt geweldig makkelijk natuurlijk en het zal misschien niet altijd precies het juiste item worden aangeklikt, maar het werkt wel enorm makkelijk. | Well, that functions incredibly easily of course, and although the selected item may not always be exactly correct, it is still very convenient to use. |  |
| 4 | Het is wel echt mijn eigen manier van notuleren. | It really is my own way of recording data. |  |
| 5 | Datgene wat je vastlegt is natuurlijk ook erg afhankelijk van wat er in je softwareprogramma zit. | That what you record is of course also very dependent on what is included in your software program. |  |
| 6 | Ik denk wel dat als je dit, zeg maar, systematisch zou willen verzamelen en dat als dat niet helemaal makkelijk geautomatiseerd kan, dat je dan wel ook veel weerstand zou krijgen. Of in ieder geval qua tijd inzet. | I do think that if you wanted to collect this systematically, and if that can’t be easily automated, then you would probably face a lot of resistance. Or at least in terms of time investment. |  |
| 7 | Ik denk dat het belangrijk is dat er hele goede instructies bij komen. | I think it is important that there are really good instructions with it. |  |
| 8 | Ja, dat zou voor mezelf wel interessant zijn om te kijken. Ben je goed bezig of zijn we op de goede weg? Of moeten we het anders gaan doen? | Yes, that would be interesting for me to look at. Are you performing well or are we on the right track? Or do we need to start doing things differently? |  |
